# Transfascial Paraspinal Versus Conventional Open Posterior Pedicle Screw Fixation for Thoracolumbar Fractures: A Randomized Controlled Trial

**DOI:** 10.7759/cureus.116169

**Published:** 2026-09-13

**Authors:** Deepak K Saini, Pooja Gunwal, Pushprajan Chauhan, Atul Sareen, Akash Yadav

**Affiliations:** 1 Department of Orthopedics, Maharishi Chyawan Government Medical College and Rao Tularam Hospital, Koriawas, IND; 2 Department of Orthopedics, Vardhman Mahavir Medical College and Safdarjung Hospital, New Delhi, IND; 3 Department of Anesthesiology, Maharishi Chyawan Government Medical College and Rao Tularam Hospital, Koriawas, IND; 4 Department of Anesthesiology, Vardhman Mahavir Medical College and Safdarjung Hospital, New Delhi, IND; 5 Department of Orthopedics, King George's Medical University, Lucknow, IND

**Keywords:** minimally invasive spine surgery, paraspinal approach, pedicle screw fixation, thoracolumbar fracture, wiltse approach

## Abstract

Introduction: Posterior pedicle screw fixation is the standard treatment for unstable thoracolumbar fractures requiring surgery. The conventional open posterior approach, however, requires extensive subperiosteal stripping of the paraspinal muscles, causing ischemia, denervation, greater intraoperative blood loss, more postoperative pain, and delayed recovery. The Wiltse paraspinal, or transfascial, approach exploits the natural intermuscular plane between the multifidus and longissimus muscles to limit this soft-tissue disruption while preserving adequate exposure. Still, robust randomized evidence comparing the two techniques remains limited. Our objective was to compare the perioperative, clinical, and radiological outcomes of pedicle screw fixation performed through the transfascial paraspinal approach versus the conventional open posterior approach in patients with AO Spine Type A thoracolumbar fractures.

Methods: Fifty patients aged 18-60 years with a single-level AO Spine Type A thoracolumbar fracture were randomized 1:1 using a computer-generated sequence with allocation concealment to transfascial paraspinal (n=25) or conventional open posterior (n=25) fixation and followed for 12 months. Outcomes included operative time, blood loss, postoperative drain output, mobilization time, hospital stay, Visual Analog Scale pain scores at two weeks and three, six, and 12 months, fluoroscopy time, sagittal Cobb angle correction, and surgical site infection. Continuous variables were compared using the independent-samples t-test or Mann-Whitney U test, and categorical variables using the chi-square or Fisher's exact test, with p<0.05 considered statistically significant.

Results: The transfascial group had significantly shorter operative time (96.16±13.77 vs. 106.64±17.70 min, p=0.047), less intraoperative blood loss (173.76±37.94 vs. 294.00±89.35 mL, p<0.001), lower drain output (37.60±18.94 vs. 93.20±43.48 mL, p<0.001), earlier mobilisation (2.44±1.04 vs. 4.08±2.77 days, p=0.033) and shorter hospital stay (3.08±1.82 vs. 5.48±4.30 days, p=0.012) than the conventional group. VAS pain scores were significantly lower in the transfascial group at two weeks, three, six, and 12 months (all p≤0.004), despite similar baseline pain (p=0.533). Postoperative Cobb angle correction (4.40±1.15° vs. 4.60±1.19°, p=0.549) and fluoroscopy time (7.04±1.35 vs. 7.68±1.65 sec, p=0.140) did not differ significantly between groups. Surgical site infection distribution was also similar (Fisher-Freeman-Halton test, p=0.349), though deep infection occurred only in the conventional group (8.0% vs. 0%).

Conclusion: The transfascial paraspinal approach achieves radiological correction equivalent to that of the conventional open posterior approach while significantly reducing operative time, blood loss, wound drainage, hospital stay, and postoperative pain. In appropriately selected patients with AO Spine Type A thoracolumbar fractures, it represents an effective, reproducible, and safe muscle-sparing alternative to conventional open fixation, supporting wider adoption pending confirmation in larger multicenter randomized trials with longer follow-up.

## Introduction

Fractures of the thoracolumbar spine are among the most common spinal injuries encountered in trauma practice, accounting for close to 60% of all vertebral fractures. The thoracolumbar junction (T10-L2) lies at the transition between the rigid thoracic cage and the more mobile lumbar spine and is therefore particularly vulnerable to high-energy trauma. Such injuries can produce spinal instability, progressive kyphosis, chronic pain, and neurological compromise, and frequently require surgery to restore alignment and mechanical stability [1-4].

Treatment selection is guided by fracture stability, neurological status, and the integrity of the posterior ligamentous complex. In neurologically intact patients, the Thoracolumbar Injury Classification and Severity Score (TLICS) is widely used to guide management by integrating injury morphology, neurological findings, and posterior ligamentous complex status [5]. When surgery is indicated, posterior pedicle screw fixation remains the technique of choice because it provides rigid three-column stabilization, aids fracture reduction, and allows early mobilization.

The conventional posterior midline approach has traditionally been the standard technique for pedicle screw instrumentation, but adequate exposure requires extensive subperiosteal stripping of the paraspinal muscles, which can cause muscle ischemia, denervation, greater intraoperative bleeding, increased postoperative pain, delayed recovery, and prolonged hospitalization [6,7]. These drawbacks have prompted the development of less invasive techniques that preserve the paraspinal musculature while maintaining adequate exposure.

The Wiltse paraspinal approach uses the natural intermuscular plane between the multifidus and longissimus to limit muscle disruption during posterior instrumentation, and has been reported to reduce blood loss, postoperative pain, and soft-tissue trauma while permitting earlier rehabilitation without compromising fixation [8-10]. Percutaneous pedicle screw fixation offers a further minimally invasive option but is limited by its dependence on fluoroscopy, greater technical complexity, need for specialized instrumentation, and concerns over screw placement accuracy [11,12].

Despite growing acceptance of minimally invasive posterior fixation, prospective randomized evidence directly comparing the transfascial paraspinal approach with the conventional open posterior approach remains scarce. This study aimed to compare the perioperative, clinical, and radiological outcomes of pedicle screw fixation performed through the transfascial paraspinal approach versus the conventional open posterior approach in patients with AO Spine Type A thoracolumbar fractures, evaluating operative time, intraoperative blood loss, postoperative drain output, fluoroscopic exposure time, time to mobilization, length of hospital stay, postoperative pain (Visual Analogue Scale), surgical site infection, and sagittal Cobb angle [13-16].

## Materials and methods

Study design and participants

This prospective randomized comparative study was conducted at a tertiary care teaching hospital over 18 months, from November 2019 to April 2021. The protocol was approved by the Institutional Review Board and Institutional Ethics Committee before enrollment on 31 October 2019, and written informed consent was obtained from every participant after the study objectives, surgical procedures, and follow-up protocol had been explained.

Patients aged 18-60 years presenting with a single-level AO Spine Type A compression fracture of the thoracolumbar junction (D10-L2) following trauma were eligible, irrespective of neurological status. Patients with AO Spine Type B or C injuries, pathological fractures, spinal tuberculosis, severe osteoporosis, multilevel vertebral fractures, previous spinal surgery, or spinal neoplasms were excluded.

Sample size estimation and randomization

Sample size was calculated from the perioperative outcome data reported by Chang et al. [17] for posterior paraspinal muscle versus conventional posterior approaches. Assuming a 5% significance level and 99% power, a minimum of 24 patients per arm was required; 50 patients were recruited, with 25 allocated to each group.

Eligible patients were randomized 1:1 to the transfascial paraspinal or conventional open posterior approach using a computer-generated sequence prepared by an investigator uninvolved in recruitment or surgical management. Allocation concealment was maintained with sequentially numbered, opaque, sealed envelopes opened only after surgery had been scheduled.

Preoperative evaluation

All patients underwent standardized preoperative clinical and radiological assessment. Standing anteroposterior and lateral radiographs were obtained to determine the sagittal Cobb angle. Computed tomography characterized fracture morphology and vertebral body comminution, and magnetic resonance imaging assessed the posterior ligamentous complex, neural elements, and soft-tissue injury. These findings guided fracture classification, operative planning, and selection of the fixation strategy.

Surgical technique

All procedures were performed under general anesthesia with the patient prone on a radiolucent operating table, with careful positioning and padding to allow a free-hanging abdomen and minimize epidural venous congestion. Pressure points, external genitalia, and the endotracheal tube were protected throughout. The affected level was confirmed with image intensification (mobile C-arm fluoroscopy unit: GE Healthcare, Brivo OEC 850, Beijing, China) before incision, and dilute adrenaline solution (1:500,000) was infiltrated into the incision, subcutaneous tissue, and paraspinal musculature to reduce bleeding. In both groups, pedicle screws and rods were placed using the spinal instrumentation system (Jayon Implants, Thoracolumbar Spinal Fixation System, Kerala, India).

Transfascial Paraspinal Approach

A midline skin incision was centered over the injured level under fluoroscopic guidance. After division of the subcutaneous tissue, the thoracolumbar fascia was exposed without extensive muscle dissection, and further adrenaline solution was infiltrated beneath the fascia (Figure 1a). Rather than opening the fascia along the full length of the skin incision, separate longitudinal fascial windows of approximately 2 cm were created 2.5 cm-7 cm lateral to the midline on each side, corresponding to the transverse process-superior articular facet junction and providing access through the intermuscular interval described by Wiltse (Figure 1b). Blunt finger dissection was used to develop the plane between the multifidus and longissimus muscles until the mammillary process or transverse process-facet junction was identified.

**Figure 1:**
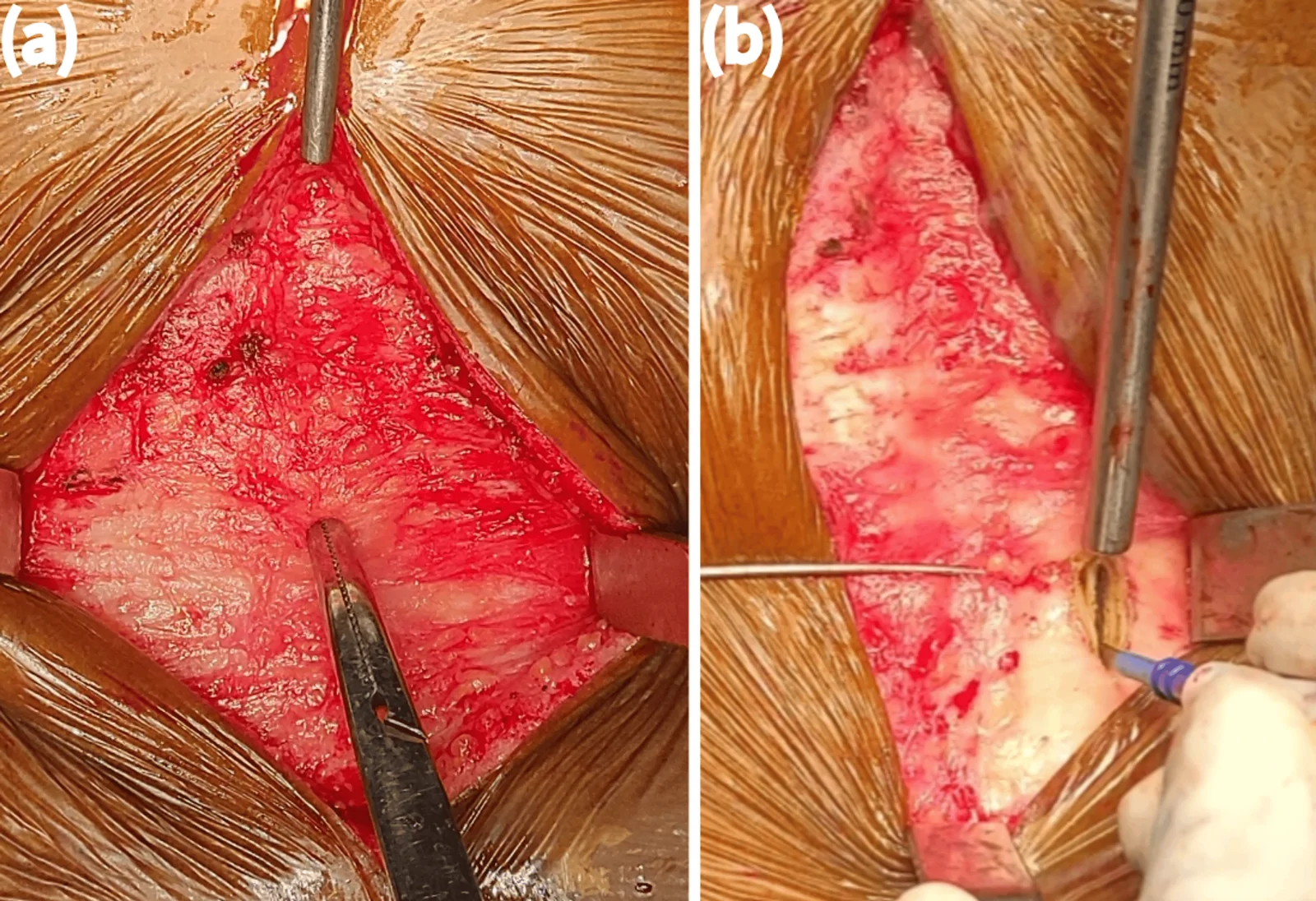
Transfascial paraspinal approach. (a) Exposure of the thoracolumbar fascia with the midline identified. (b) Longitudinal paraspinal fascial incision

A small vascular bundle supplying the pars interarticularis was frequently encountered (Figure 2a) and served as a useful landmark for localizing the pedicle entry point (Figure 2b) [9].

**Figure 2:**
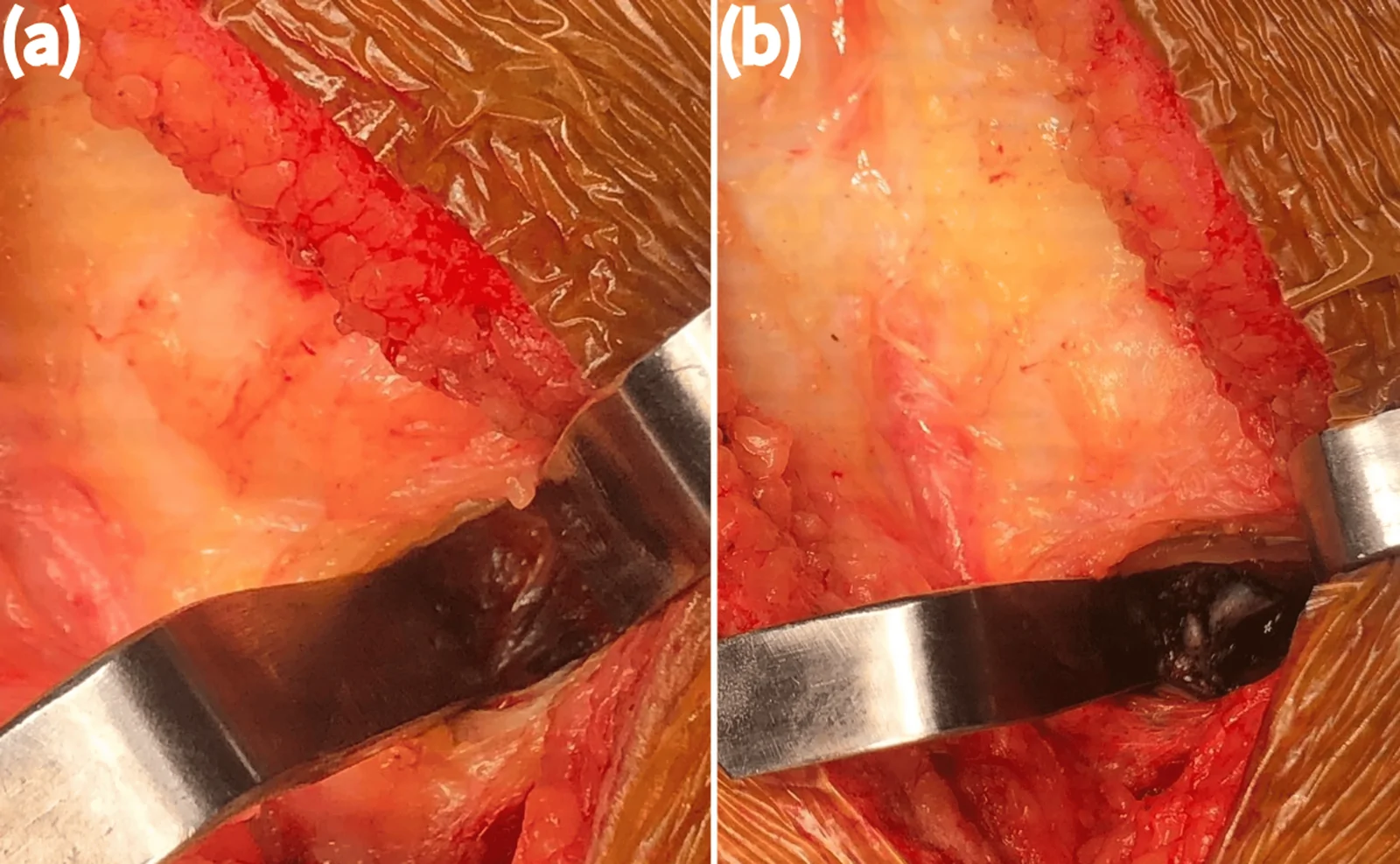
(a) Perforator vessels visible within the paraspinal plane, used as an anatomical landmark. (b) Pedicle entry point identified immediately beneath the vascular leash

Narrow right-angled or tubular retractors maintained exposure while minimizing pressure-related muscle injury. After clearing the soft tissue overlying the entry point with electrocautery, its position was confirmed fluoroscopically. Pedicle screws were inserted freehand: a cortical opening was made with an awl, the pedicle channel prepared sequentially with curved probes, and the superior, inferior, medial, and lateral pedicle walls checked with a ball-tipped probe before definitive screw placement (Figure 3).

**Figure 3:**
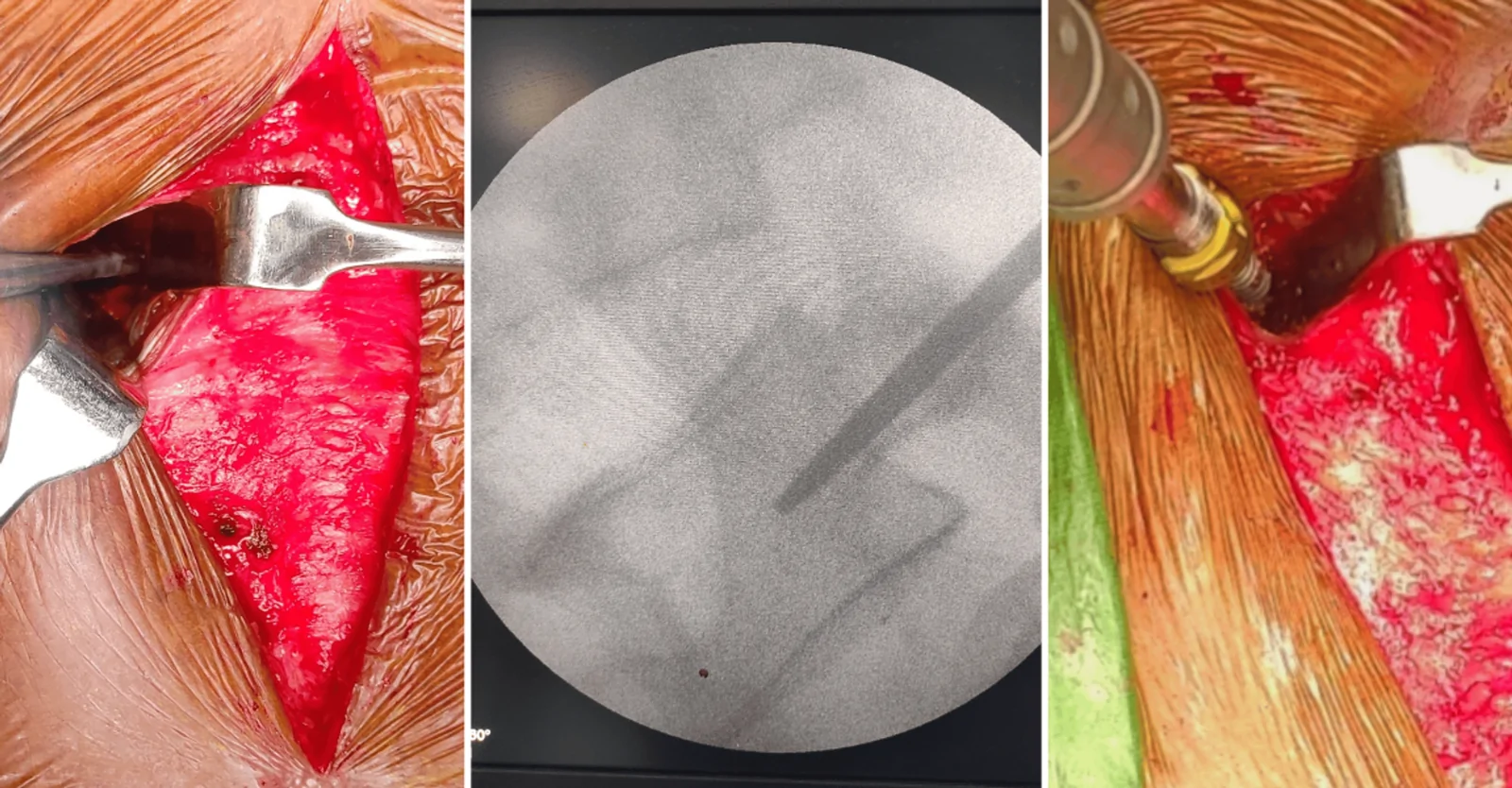
Pedicle screws inserted through the paraspinal window using the freehand technique

When neural decompression was required, adjacent fascial windows were connected if wider exposure was needed, preserving muscle attachment as far as possible (Figure 4a). Decompression was performed by laminotomy, limited facetectomy, or a transpedicular corridor according to fracture morphology, with retropulsed bone fragments repositioned using a bone punch (Figure 4b). 

**Figure 4:**
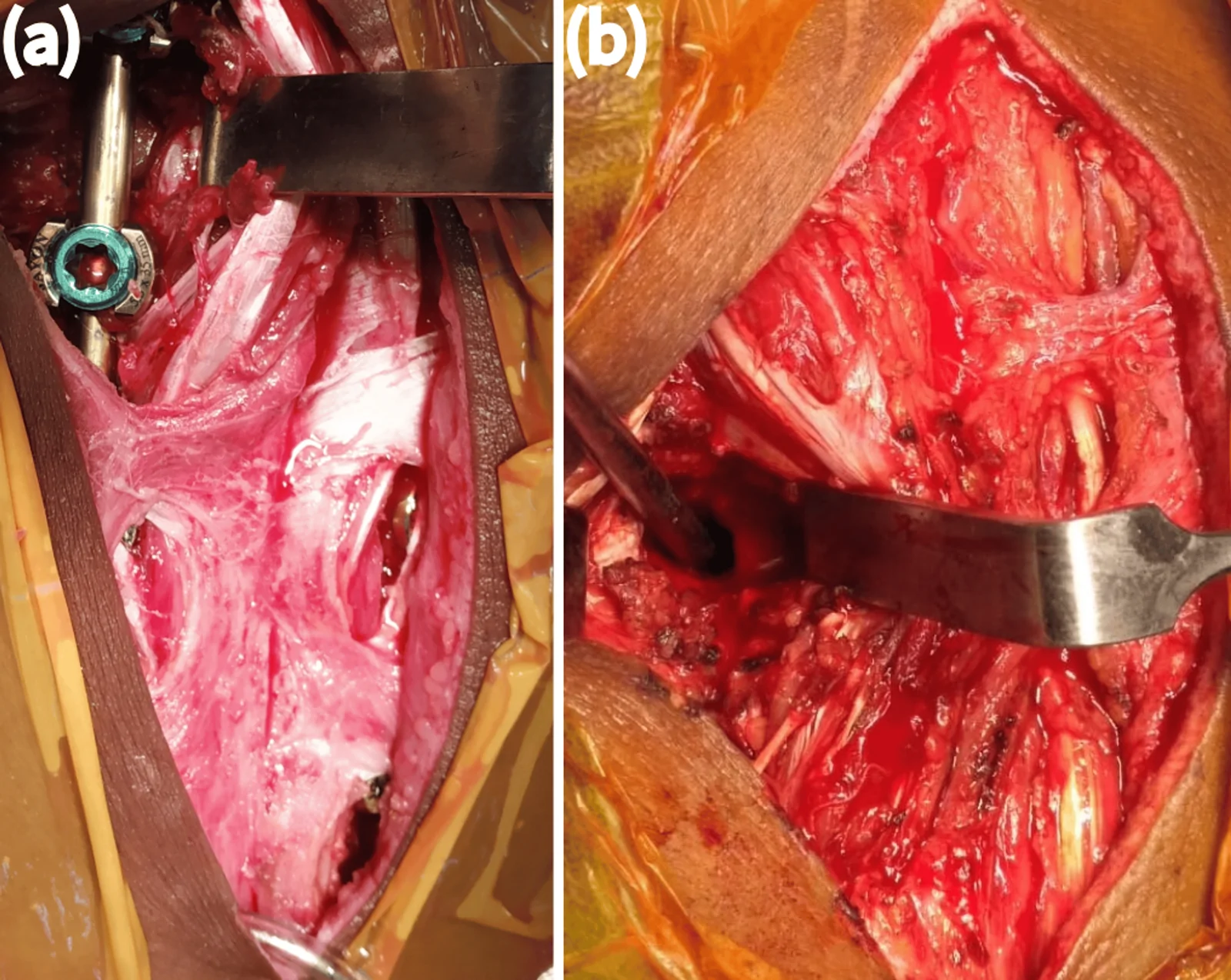
(a) Adjacent fascial windows connected to widen exposure for decompression. (b) Transpedicular decompression of a retropulsed osseous fragment

Additional indirect decompression was achieved by controlled distraction and ligamentotaxis after screw placement. Contoured rods were then inserted, secured with locking caps, and the construct tightened (Figure 5a). The paraspinal fascia at each window was closed in layers (Figure 5b), and postoperative anteroposterior and lateral radiographs confirmed satisfactory implant position (Figure 5c).

**Figure 5:**
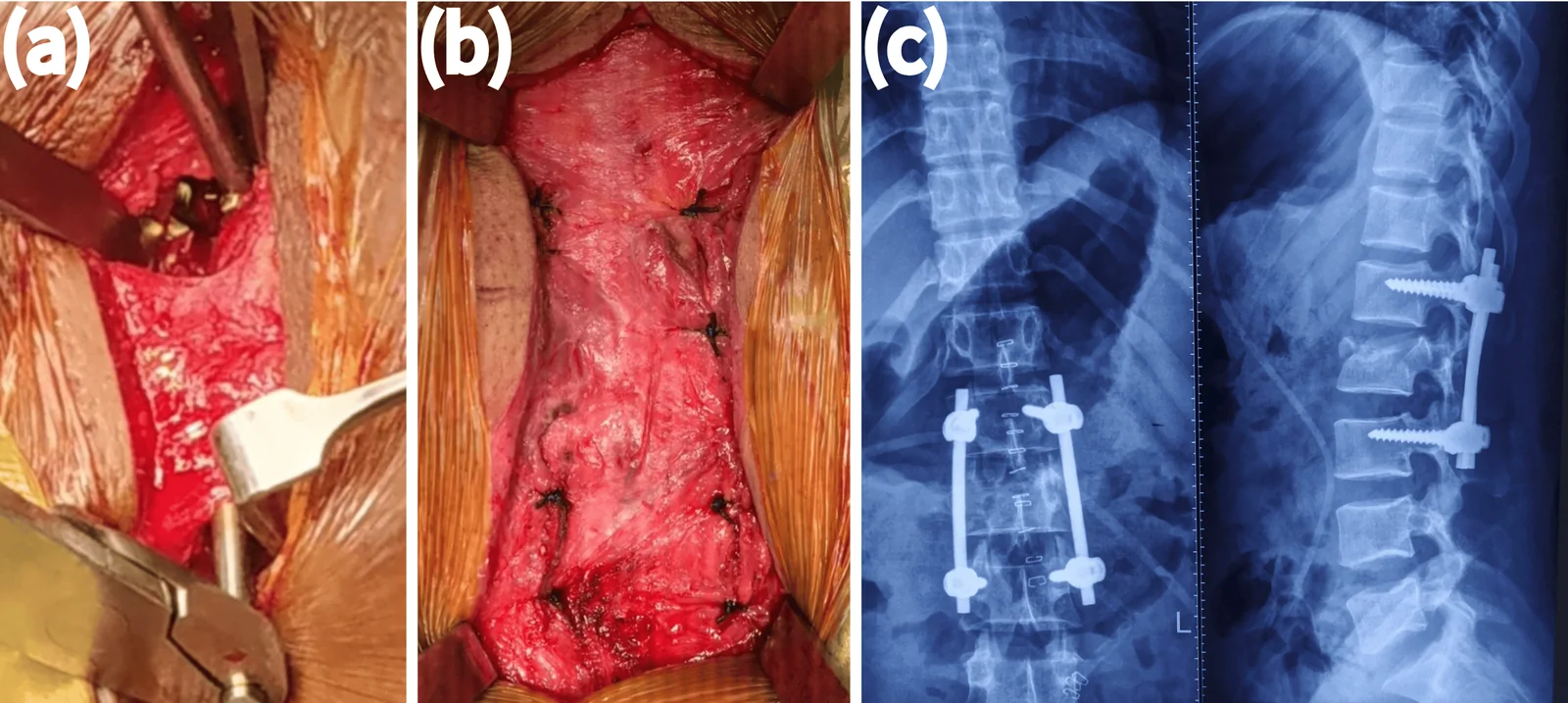
(a) Contoured rods inserted and secured. (b) Closure of the paraspinal fascia. (c) Postoperative radiograph confirming implant position

Conventional Posterior Open Approach

For the conventional technique, a standard posterior midline incision was made over the involved segment. After fluoroscopic confirmation of the level, the thoracolumbar fascia was incised in the midline, and the paraspinal muscles were elevated subperiosteally from the spinous processes, laminae, and transverse processes with electrocautery and periosteal elevators to fully expose the posterior bony elements (Figure 6a). Pedicle screws were placed freehand, followed by application of longitudinal rods secured with locking caps. Decompression, where indicated, was performed by laminotomy or laminectomy according to intraoperative findings (Figure 6b).

**Figure 6:**
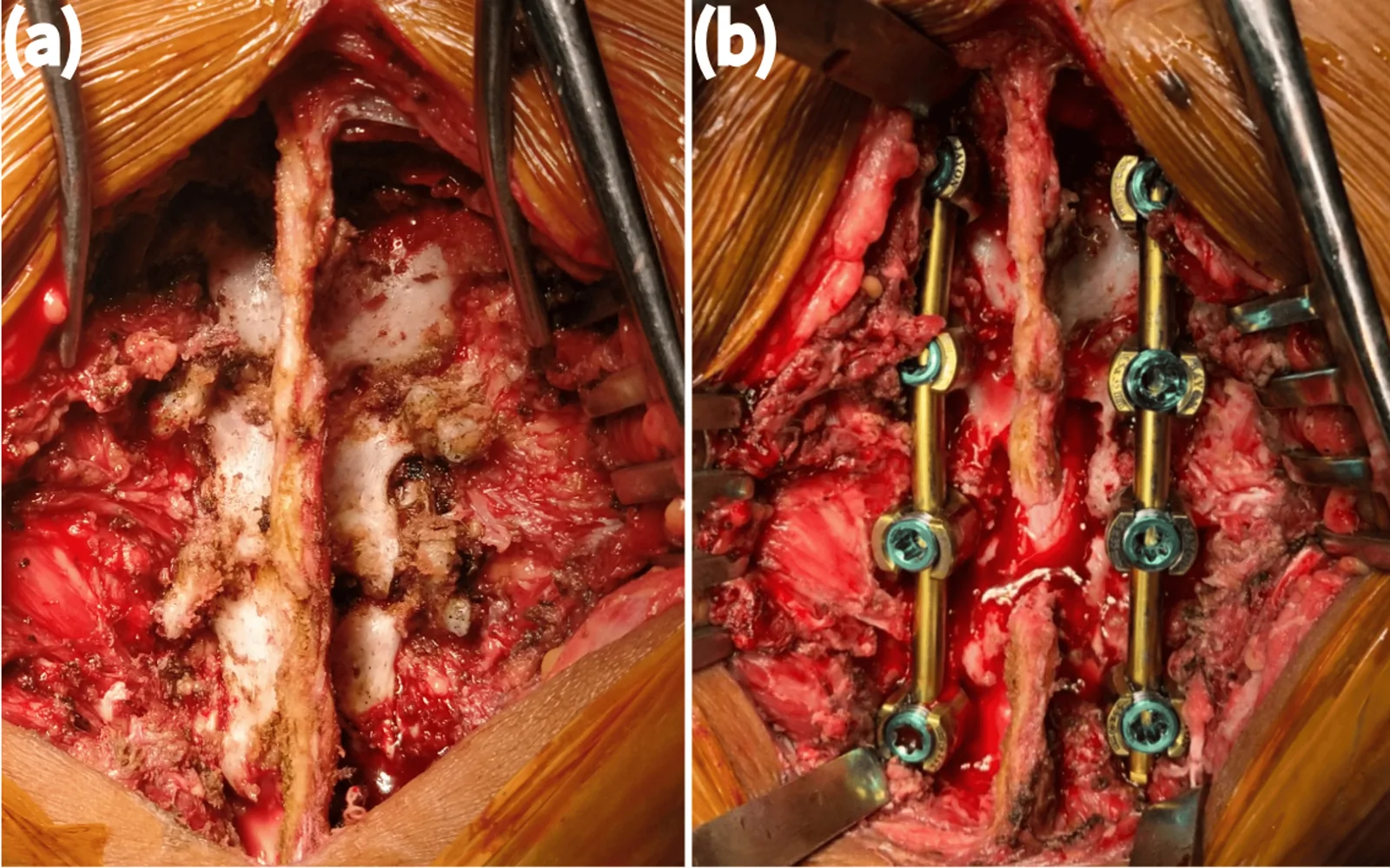
Conventional posterior open approach. (a) Subperiosteal dissection and elevation of the paraspinal muscles. (b) Pedicle screw fixation with neural decompression

In both groups, the operative field was irrigated thoroughly with normal saline. A closed-suction drain was placed, meticulous layered wound closure was performed, and a sterile dressing was applied before the patient was transferred to recovery.

Outcome measures

Primary outcomes were operative time, estimated intraoperative blood loss, fluoroscopic exposure time, drain output during the first 24 hours, time to independent mobilization, hospital stay, postoperative pain, surgical site infection, and radiological correction. Pain was assessed with a 10-point Visual Analogue Scale preoperatively and at two weeks and three, six, and 12 months postoperatively. Standard anteroposterior and lateral radiographs were obtained postoperatively to verify implant position and determine the sagittal Cobb angle. Clinical, perioperative, and radiological outcomes were compared between the two groups.

Statistical analysis

Data were analyzed using IBM SPSS Statistics version 21.0 (IBM Corp., Armonk, NY, USA). Categorical variables were summarized as frequencies and percentages, and continuous variables as mean ± standard deviation or median (interquartile range) according to distribution, assessed with the Shapiro-Wilk test. Between-group comparisons used the independent-samples t-test for normally distributed variables and the Mann-Whitney U test otherwise; categorical variables were compared with the chi-square or Fisher's exact test as appropriate. All tests were two-sided, with p<0.05 considered statistically significant.

## Results

Of 65 patients assessed for eligibility, 15 were excluded (6 with AO Spine Type B or C injuries, four with pathological fractures, and five who declined participation). Fifty patients were randomized, 25 to the transfascial paraspinal approach and 25 to the conventional open posterior approach; all received the allocated intervention, completed the 12-month follow-up, and were included in the final analysis (Figure 7).

**Figure 7:**
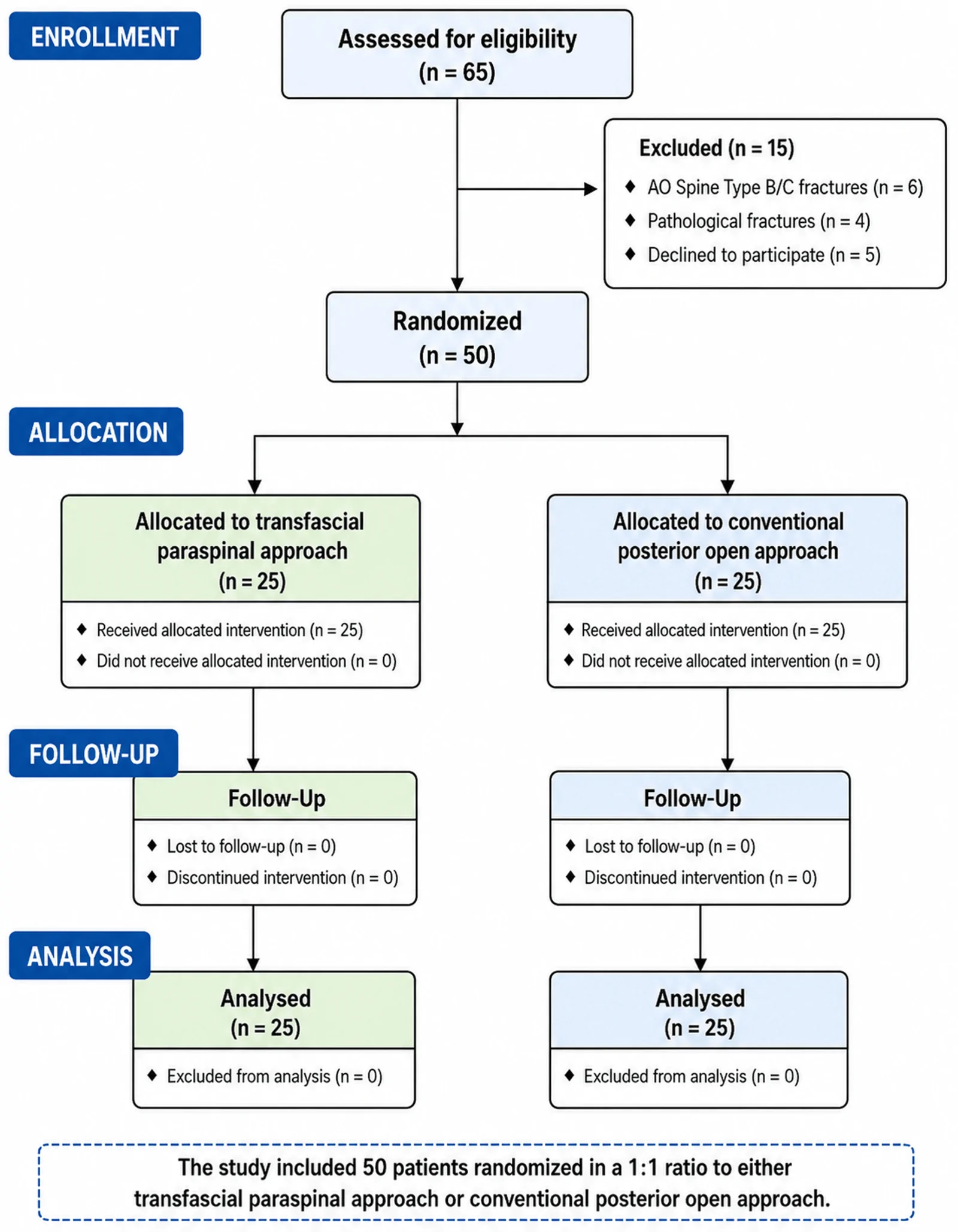
CONSORT 2010 flow diagram summarizing patient enrollment, allocation, follow-up, and analysis CONSORT, Consolidated Standards of Reporting Trials

Demographic and clinical profile 

The cohort was predominantly male (80%, n=40); the remaining 20% (n=10) were female. Fall from height was the commonest mechanism of injury (64%), followed by road traffic accidents (22%), fall from stairs (8%), assault (4%), and overhead fall (2%). Most fractures were AO Spine Type A4 (60%), followed by Type A3 (32%) and Type A2 (8%); L1 was the most frequently injured level (30%) (Table 1).

**Table 1:** Demographic and clinical profile of the study cohort Data are presented as number (%). AO, Arbeitsgemeinschaft für Osteosynthesefragen; D, thoracic vertebra; L, lumbar vertebra

Characteristic	Category	n (%)
Sex	Male	40 (80)
	Female	10 (20)
Mechanism of injury	Assault	2 (4)
	Fall from height	32 (64)
	Overhead fall	1 (2)
	Fall from stairs	4 (8)
	Road traffic accident	11 (22)
Type of fracture (AO Spine classification)	A2	4 (8)
	A3	16 (32)
	A4	30 (60)
Level of fracture	D10	6 (12)
	D11	9 (18)
	D12	13 (26)
	L1	15 (30)
	L2	7 (14)

Comparison of perioperative and clinical outcomes

Intraoperative and early postoperative outcomes favored the transfascial paraspinal approach. Mean operative time was shorter in the transfascial group than in the open group (96.16±13.77 vs. 106.64±17.70 minutes; t=-2.337, p=0.047), and intraoperative blood loss was markedly lower (173.76±37.94 vs. 294.00±89.35 mL; t=-6.193, p<0.001). Drain output on postoperative day 1 was also significantly lower after the transfascial approach (37.60±18.94 vs. 93.20±43.48 mL; t=-5.862, p<0.001).

Patients in the transfascial group mobilized significantly earlier (2.44±1.04 vs. 4.08±2.77 days; t=-2.771, p=0.033) and had a shorter hospital stay (3.08±1.82 vs. 5.48±4.30 days; t=-2.654, p=0.012) (Table 2).

**Table 2:** Comparison of perioperative, clinical, and radiological outcomes between groups Data are presented as mean ± SD. Continuous variables were compared using the independent-samples t-test or Mann-Whitney U test, as appropriate, according to data distribution. The corresponding t or U statistic is reported for each variable. A two-tailed P-value <0.05 was considered statistically significant. VAS, visual analogue scale; POD, postoperative day

Variable	Transfascial (n=25), mean±SD	Conventional open (n=25), mean±SD	t statistic	P-value
Operative time (min)	96.16±13.77	106.64±17.70	-2.337	0.047
Intraoperative blood loss (mL)	173.76±37.94	294.00±89.35	-6.193	<0.001
Drain output, POD-1 (mL)	37.60±18.94	93.20±43.48	-5.862	<0.001
Mobilisation time (days)	2.44±1.04	4.08±2.77	-2.771	0.033
Hospital stay (days)	3.08±1.82	5.48±4.30	-2.654	0.012
VAS score, preoperative	6.80±0.96	6.96±0.84	-0.628	0.533
VAS score, 2 weeks	5.32±0.80	6.28±1.08	-3.564	<0.001
VAS score, 3 months	4.02±0.52	5.14±0.98	-5.05	<0.001
VAS score, 6 months	2.66±0.74	3.42±0.40	-4.52	<0.001
VAS score, 12 months	1.28±0.46	1.84±0.71	-3.310	0.004
Cobb angle, preoperative (°)	10.56±1.94	10.44±2.00	0.215	0.83
Cobb angle, POD-15 (°)	4.40±1.15	4.60±1.19	-0.603	0.549
Fluoroscopy exposure time (sec)	7.04±1.35	7.68±1.65	-1.50	0.14

Preoperative VAS pain scores were comparable between groups (6.80±0.96 vs. 6.96±0.84; t =-0.628, p=0.533), confirming similar baseline pain. Postoperative pain improved significantly more in the transfascial group throughout follow-up: at two weeks (5.32±0.80 vs. 6.28±1.08; t=-3.564, p<0.001), three months (4.02±0.52 vs. 5.14±0.98; t=-5.05, p<0.001), and six months (2.66±0.74 vs. 3.42±0.40; t =-4.52, p<0.001). At 12 months, both groups had substantial pain relief, but VAS scores remained significantly lower in the transfascial group (1.28±0.46 vs. 1.84±0.71; t=-3.310, p=0.004).

Both techniques achieved comparable sagittal correction. Preoperative Cobb angle did not differ significantly between groups (10.56±1.94° vs. 10.44±2.00°; t=0.215, p=0.830), nor did the angle measured on postoperative day 15 (4.40±1.15° vs. 4.60±1.19°; t=-0.603, p=0.549). Mean fluoroscopic exposure time was slightly, but not significantly, lower in the transfascial group (7.04±1.35 vs. 7.68±1.65 seconds; t=-1.50, p=0.140).

Surgical site infection 

Surgical site infection was uncommon overall. One patient (4.0%) in the transfascial group developed a superficial infection, with no deep infections. In the conventional open group, one patient (4.0%) developed a superficial infection, and two (8.0%) developed deep infections. Although infection was numerically more frequent after the open approach, the distribution of infection severity did not differ significantly between groups (Fisher-Freeman-Halton exact test, p=0.349) (Table 3).

**Table 3:** Distribution of surgical site infection between groups Data are presented as number (%). P=0.349 for the overall distribution of infection severity between groups (Fisher-Freeman-Halton exact test). A p-value <0.05 was considered statistically significant.

Surgical site infection	Transfascial (n=25)	Conventional open (n=25)	Total (n=50)
Deep infection	0 (0.0%)	2 (8.0%)	2 (4.0%)
Superficial infection	1 (4.0%)	1 (4.0%)	2 (4.0%)
No infection	24 (96.0%)	22 (88.0%)	46 (92.0%)
Total	25 (100%)	25 (100%)	50 (100%)

## Discussion

The principal finding of this randomized trial is that pedicle screw fixation through a transfascial paraspinal approach achieves radiological correction equivalent to the conventional open posterior approach while providing clinically meaningful perioperative advantages. Patients treated with the muscle-sparing technique had shorter operative time, less blood loss, reduced drainage, earlier mobilization, shorter hospitalization, and better postoperative pain relief, without compromising deformity correction or increasing fluoroscopic exposure.

The shorter operative time and lower blood loss observed with the transfascial technique are biologically plausible: the Wiltse intermuscular interval provides direct access to the posterior elements without extensive muscle detachment, preserving normal tissue planes and limiting disruption of the paraspinal vascular supply. Comparable reductions in operative time and blood loss have been reported by Li et al. and Chang et al., supporting the reproducibility of these benefits across different populations and settings [17,18].

The lower drain output in the transfascial group further supports reduced soft-tissue injury with this approach: limited muscle stripping decreases dead space and preserves local vascularity, both of which limit postoperative wound drainage. Similar findings have been reported in other studies of muscle-preserving posterior approaches [17,18].

Faster mobilization and shorter hospital stay in the transfascial group likely reflect the combined effects of less muscle disruption, lower pain, reduced drainage, and lower physiological stress. Earlier mobilization is particularly relevant after spinal trauma because it supports rehabilitation and may reduce complications of prolonged bed rest, consistent with previous reports of paraspinal muscle-sparing techniques [17,18].

Baseline pain scores were similar between groups, confirming comparable preoperative status, but patients treated through the transfascial approach reported significantly lower pain throughout follow-up. Preservation of the posterior musculature, avoiding extensive subperiosteal stripping, probably limits muscle ischemia, denervation, and scarring, contributing to sustained pain relief - an observation also made by Li et al. and Chang et al., who attributed better functional recovery to preservation of the paraspinal muscles and posterior tension band [17,18].

Comparable postoperative Cobb angles indicate that the transfascial approach achieves fracture reduction and sagittal alignment equivalent to the conventional approach, confirming that minimizing surgical exposure does not compromise the biomechanical goals of posterior pedicle screw fixation, consistent with previous comparative studies [17,18].

A recognized concern with muscle-sparing techniques is greater reliance on fluoroscopy. In this study, fluoroscopic exposure time was similar between groups, indicating that the transfascial approach did not increase radiation exposure; the recorded times also compare favorably with those reported for percutaneous pedicle screw fixation, suggesting that the technique combines limited soft-tissue dissection with acceptable imaging requirements [2,12].

Although the difference was not statistically significant, deep infection occurred only after the conventional open approach. The study was not powered to detect differences in uncommon complications, but this trend is clinically relevant: preserved muscle vascularity, smaller dissection, and reduced dead space with the transfascial approach may lower the risk of wound complications, an observation also suggested in previous reports of less invasive posterior procedures.

Although surgical exposure differs considerably between the techniques, adequate neural decompression is achievable with either approach when appropriately indicated. In the transfascial technique, decompression can be performed by laminotomy, partial facetectomy, or a transpedicular corridor, with adjacent fascial windows connected when wider exposure is required, whereas the conventional approach provides unrestricted visualization through standard posterior exposure. Despite the steeper learning curve of the transfascial approach, our findings show that satisfactory decompression and stable instrumentation can be achieved while preserving the posterior musculoligamentous envelope.

These findings should be weighed against the strengths and limitations of the study design. Its prospective randomized design minimized selection bias and allowed direct comparison of the two approaches, and allocation concealment using sequentially numbered, opaque envelopes strengthened internal validity; baseline demographic, clinical, and radiological characteristics were comparable between groups, and all procedures were performed with a standardized technique at a single tertiary center, reducing variability. Complete follow-up of all enrolled patients eliminated attrition bias, and a broad range of clinically relevant perioperative, radiological, and postoperative outcomes was assessed.

At the same time, several limitations warrant consideration. The study was conducted at a single tertiary center with a relatively small sample size, limiting external validity, and the 12-month follow-up, although adequate for early clinical and radiological outcomes, was insufficient to assess long-term implant performance, maintenance of alignment, adjacent segment degeneration, or delayed complications. The transfascial approach is technically demanding and requires familiarity with the Wiltse plane, so the learning curve may affect operative efficiency early in adoption, and exposure through limited fascial windows may be more challenging in obese patients. Because only AO Spine Type A fractures were included, the findings cannot be generalized to Type B or C injuries, which often need more extensive exposure for reduction, decompression, and stabilization, and the study was not powered to detect differences in relatively uncommon outcomes such as surgical site infection. Blinding of surgeons and patients was not feasible given the nature of the intervention, introducing potential performance bias, although standardized protocols and objective outcome measures limited assessment bias; validated patient-reported outcome measures such as the Oswestry Disability Index and Short Form-36 were also not assessed, limiting evaluation of functional recovery and health-related quality of life.

## Conclusions

In appropriately selected patients with AO Spine Type A thoracolumbar fractures, the transfascial paraspinal approach achieves pedicle screw fixation and sagittal correction equivalent to the conventional open posterior approach, while significantly reducing operative time, blood loss, wound drainage, hospital stay, and postoperative pain.

Take-home message for surgeons. When anatomy and experience allow, preserving the natural paraspinal muscle plane through the transfascial window is a safe and effective muscle-sparing alternative to conventional open fixation, delivering the same fixation stability with a faster, less painful recovery for the patient. Confirmation in larger multicenter trials with longer follow-up is warranted before wider adoption.

## References

[1] Magerl F, Aebi M, Gertzbein SD, Harms J, Nazarian S (1994). A comprehensive classification of thoracic and lumbar injuries. Eur Spine J.

[2] Zhang W, Li H, Zhou Y (2016). Minimally invasive posterior decompression combined with percutaneous pedicle screw fixation for the treatment of thoracolumbar fractures with neurological deficits: a prospective randomized study versus traditional open posterior surgery. Spine (Phila Pa 1976).

[3] Garg B, Dixit V, Batra S, Malhotra R, Sharan A (2017). Non-surgical management of acute osteoporotic vertebral compression fracture: a review. J Clin Orthop Trauma.

[4] Kumar N, Osman A, Chowdhury JR (2017). Traumatic spinal cord injuries. J Clin Orthop Trauma.

[5] Vaccaro AR, Lehman RA Jr, Hurlbert RJ (2005). A new classification of thoracolumbar injuries: the importance of injury morphology, the integrity of the posterior ligamentous complex, and neurologic status. Spine (Phila Pa 1976).

[6] Min SH, Kim MH, Seo JB, Lee JY, Lee DH (2009). The quantitative analysis of back muscle degeneration after posterior lumbar fusion: comparison of minimally invasive and conventional open surgery. Asian Spine J.

[7] Sihvonen T, Herno A, Paljärvi L, Airaksinen O, Partanen J, Tapaninaho A (1993). Local denervation atrophy of paraspinal muscles in postoperative failed back syndrome. Spine (Phila Pa 1976).

[8] Wiltse LL, Bateman JG, Hutchinson RH, Nelson WE (1968). The paraspinal sacrospinalis-splitting approach to the lumbar spine. J Bone Joint Surg Am.

[9] Vialle R, Court C, Khouri N (2005). Anatomical study of the paraspinal approach to the lumbar spine. Eur Spine J.

[10] Vialle R, Wicart P, Drain O, Dubousset J, Court C (2006). The Wiltse paraspinal approach to the lumbar spine revisited: an anatomic study. Clin Orthop Relat Res.

[11] Magerl FP (1984). Stabilization of the lower thoracic and lumbar spine with external skeletal fixation. Clin Orthop Relat Res.

[12] Ikeuchi H, Ikuta K (2016). Accuracy of pedicle screw insertion in the thoracic and lumbar spine: a comparative study between percutaneous screw insertion and conventional open technique. Arch Orthop Trauma Surg.

[13] Vaccaro AR, Oner C, Kepler CK (2013). AOSpine thoracolumbar spine injury classification system: fracture description, neurological status, and key modifiers. Spine (Phila Pa 1976).

[14] Heller GZ, Manuguerra M, Chow R (2016). How to analyze the visual analogue scale: myths, truths and clinical relevance. Scand J Pain.

[15] Chiarotto A, Maxwell LJ, Ostelo RW, Boers M, Tugwell P, Terwee CB (2019). Measurement properties of visual analogue scale, numeric rating scale, and pain severity subscale of the Brief Pain Inventory in patients with low back pain: a systematic review. J Pain.

[16] Wang J, Zhang J, Xu R, Chen TG, Zhou KS, Zhang HH (2018). Measurement of scoliosis Cobb angle by end vertebra tilt angle method. J Orthop Surg Res.

[17] Chang W, Zhang D, Liu W, Lian X, Jiao Z, Chen W (2018). Posterior paraspinal muscle versus post-middle approach for the treatment of thoracolumbar burst fractures: a randomized controlled trial. Medicine (Baltimore).

[18] Li H, Yang L, Xie H, Yu L, Wei H, Cao X (2015). Surgical outcomes of mini-open Wiltse approach and conventional open approach in patients with single-segment thoracolumbar fractures without neurologic injury. J Biomed Res.

